# Tumor-targeting *Salmonella typhimurium* A1-R arrests growth of breast-cancer brain metastasis

**DOI:** 10.18632/oncotarget.2811

**Published:** 2014-11-15

**Authors:** Yong Zhang, Shinji Miwa, Nan Zhang, Robert M. Hoffman, Ming Zhao

**Affiliations:** ^1^ AntiCancer, Inc., San Diego, CA, USA; ^2^ Department of Surgery, University of California San Diego, San Diego, CA, USA

**Keywords:** *Salmonella typhimurium* A1-R, breast cancer, 4T1, brain metastasis, orthotopic

## Abstract

Brain metastasis is a morbid, treatment-resistant, end-stage frequent occurrence in breast cancer patients. The aim of this study was to evaluate the efficacy of tumor-targeting *Salmonella typhimurium* A1-R on breast cancer brain metastases. High brain-metastatic variants of murine 4T1 breast cancer cells expressing red fluorescent protein (RFP) were injected orthotopically in the mammary fat pad in non-transgenic nude mice or in the left ventricle of non-transgenic nude mice and transgenic nude mice expressing nestin-driven green fluorescent protein (ND-GFP). ND-GFP mice express GFP in nascent blood vessels. In the orthotopically-injected mice, the primary tumor was surgically-resected in order to allow brain metastasis to develop. At various time points, the tumors and vasculature in the brain were imaged by confocal and stereo fluorescence microscopy. Some of the breast cancer cells that reached the brain extravasated and grew perivascularly and some of the cells proliferated within the vasculature. *S. typhimurium* A1-R significantly inhibited brain metastasis in both metastatic models and increased survival of the orthotopically-transplanted, primary-tumor-resected mice (p<0.05). The results of the present study suggest the clinical potential of bacterial therapy of breast cancer brain metastasis.

## INTRODUCTION

Records for > 200 years have documented cancer patients going into remission after a bacterial infection. In the late nineteenth century, William B. Coley at New York Cancer Hospital, the precursor of Sloan-Kettering Memorial Cancer Center, treated cancer patients with *S. pyogenes*. In 1891, Coley noted that a sarcoma patient had tumor regression after an infection with *S. pyogenes*. Coley's first patient infected with *S. pyogenes* recovered from head and neck cancer. Coley injected many cancer patients with *S. pyogenes* and often had good results. Because of the danger of live streptococcal organisms, Coley subsequently used killed *S. pyogenes* with a second killed organism now known as *Serratia marcescens*. These killed organisms became known as Coley's Toxins [1].

Bacterial therapy of cancer has previously used anaerobic bacteria, for example *Bifidobacterium* [2] and *Clostridium* [3], which replicate only in necrotic areas of tumors. Anaerobic bacteria cannot grow in viable tumor tissue, which limits their efficacy. Anaerobic bacteria must be used in combination with chemotherapy to be effective [4, 5]. Yazawa et al. observed that *Bifidobacterium longum*, which is an obligate anaerobe, selectively localized to and proliferated in chemically-induced rat mammary tumors after systemic administration [2]. *Clostridium novyi*, with its lethal toxin removed (*C. novyi* no toxin [NT]), was generated. When *C. novyi*-NT spores were administered in combination with chemotherapy, hemorrhagic necrosis of tumors developed and the tumors regressed [3]. The disadvantage of the obligate anaerobes described above is that they do not grow in viable regions of tumors due to high oxygen tension. *C. novyi*-NT apparently has to be injected intravutumorally (i.t.). possibly precluding targeting metastasis. However, i.t. injection of *C. novyi*-NT has been shown to be effective against primary tumors in dogs and against a patient with a leomysarcoma where one tumor deposit regressed after direct i.t. injection of *C.novyi*-NT [6].

A multi-attenuated strain *S. typhimurium,* a facultative anaerobe that can grow with or without oxygen, *has been evaluated in a Phase I* clinical trial [7]. We have developed the genetically-modified *Salmonella typhimurium* A1-R (*S. typhimurium* A1-R) strain that selectively targets tumors [8-22]. *S. typhimurium* A1-R is auxotrophic for Leu and Arg, which precludes it from growing continuously in normal tissues but allows high tumor virulence. *S. typhimurium* A1-R eradicated or inhibited primary and metastatic tumors as monotherapy in nude mice with prostate [8, 10], breast [9], lung [19] and pancreatic cancers [11-13], including pancreatic cancer stem cells [14] and pancreatic cancer patient-derived orthotopic xenograft [PDOX] models [15], as well as sarcoma [12, 18] and glioma [16, 22]. Nude mice with MDA-MB-435 human breast cancer, expressing red fluorescent protein (RFP), were treated with *S. typhimurium* A1-R by three routes; p.o., i.v. and i.t. *S. typhimurium* A1-R targeted tumors at much higher levels than normal organs after all three routes of administration. The fewest bacteria were detected in normal organs after p.o. administration. The i.v. route had the greatest antitumor efficacy. There were no obvious toxic effects on the host with any of the routes of administration [20]. One mechanism of the antitumor efficacy of *S. typhimurium* is tumor blood vessel destruction [17, 23].

Bone metastasis is a lethal and morbid late stage of breast cancer that is currently treatment resistant. Treatment with *S. typhimurium* A1-R completely prevented the appearance of bone metastasis of a high-metastatic breast-cancer variant in nude mice [21].

In the present study, we demonstrated that *S. typhimurium* A1-R could inhibit breast-cancer brain metastasis, a highly lethal aspect of this disease.

## RESULTS

### Analysis of brain metastasis

**Figure 1:**
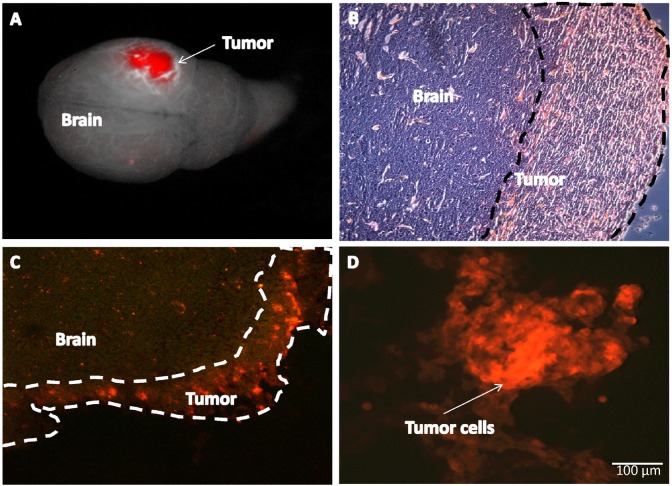
Brain metastasis of RFP 4T1 in nude mice A: 4T1 RFP brain metastasis in mice two weeks after resection of the primary tumor. Images were obtained with the Maestro fluorescence imaging system. B: Metastatic breast tumor in the brain cortex (H&E stain) (magnification 200x). C: Frozen sections were observed with an IV-100 scanning laser microscope equipped with 488 nm argon laser (Olympus Corp, Tokyo, Japan). D: 4T1-RFP breast cancer cells were isolated from brain metastasis for further rounds of *in vivo* selection of brain metastasis. Cells observed *in vitro* with an IX71 inverted microscope (Olympus) (Bar = 100 μm).

**Figure 2:**
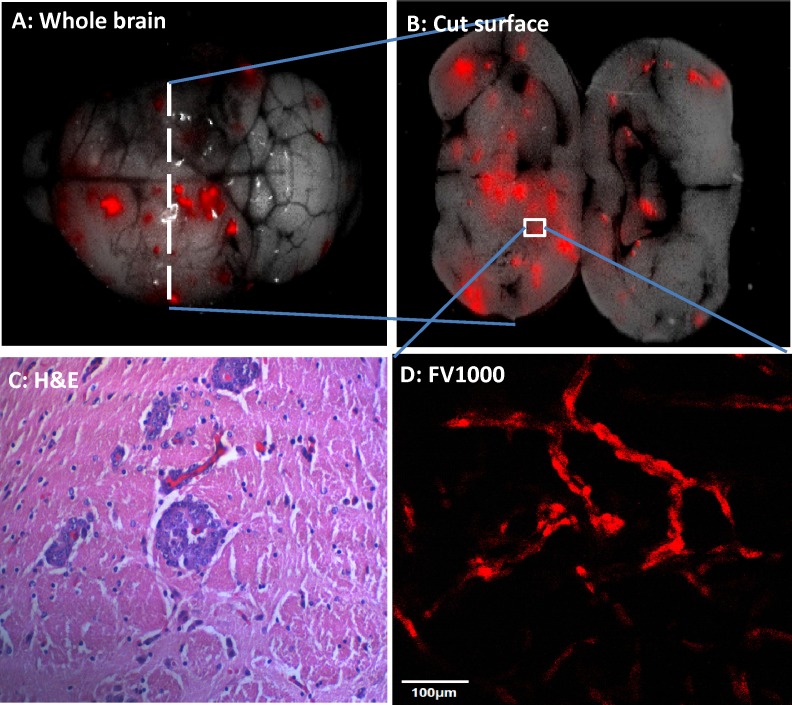
Brain metastasis after cardiac ventrical injection of high brain-metastatic variants 4T1-RFP cells were injected into the left cardiac ventricle of female nude mice. (A) Intact brain with metastases. (B) Metastases were located in both cerebral hemispheres. (C) H&E stain demonstrates thrombosis-like infarction of the brain parenchyma (agnification 200x). (D) Intravascular metastases appears to follow blood vessels (confocal fluorescence microscopy). Bar = 100 μm.

**Figure 3:**
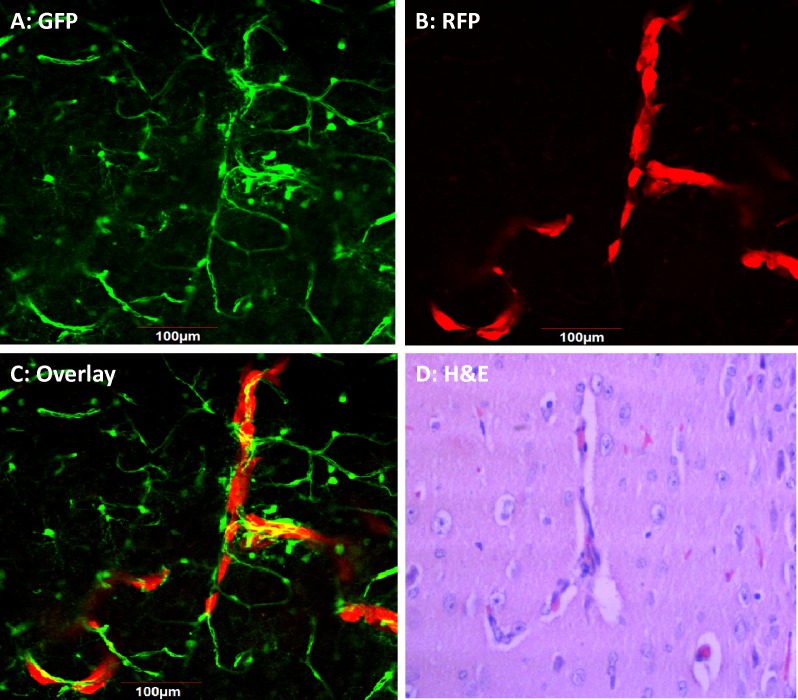
Intravascular growth of breast cancer brain metastasis in nestin-driven GFP (ND-GFP) nude mice 4T1-RFP cells were injected into the left cardiac ventricle of female ND-GFP nude mice. Intact brains were recovered from mice on days 7-20 after injection. (A) ND-GFP-expressing blood vessels. (B) RFP-expressing metastases. (C) Merged image of GFP blood vessels and RFP metastases. (A-C) Confocal fluorescence microscopy. (D) H&E staining of a thin-section (from the area of C).

4T1-RFP high-brain-metastatic variant breast cancer cells were obtained by 4 cycles of in vivo section. Upon orthotopic implantation of high metastatic 4T1-RFP variant, brain metastasis was found 2 weeks after primary tumor resection. Images were obtained with the Maestro fluorescence imaging system (Figure 1). Intact brains were recovered from mice on days 7-20 after injection. After injection of high-metastatic variants into the left cardiac ventricle, metastases were located in both cerebral hemispheres (Figure 2). H&E staining demonstrated thrombosis-like infarction of the brain parenchyma. Brain metastases appeared to grow within and around GFP-expression blood vessels (Figure 3). In ND-GFP mice, the cancer cells, expressing very bright RFP fluorescence, appeared to be inside the blood vessels which had very bright GFP fluorescence (Figure 3).

### Whole-body imaging of the efficacy of *S. typhimurium* A1-R on the growth of 4T1-RFP brain metastases

**Figure 4:**
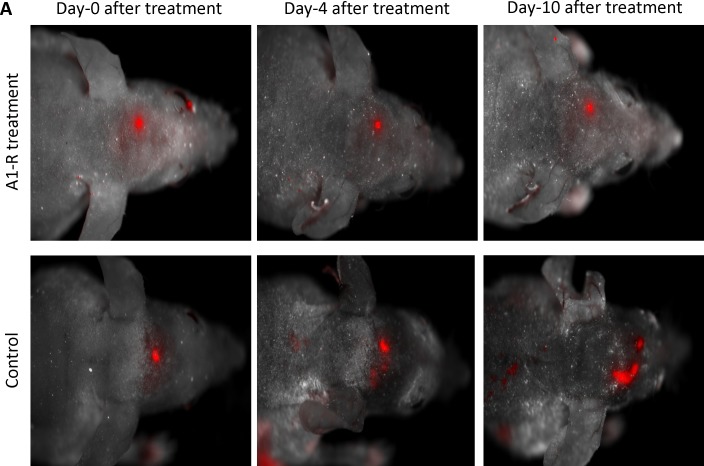

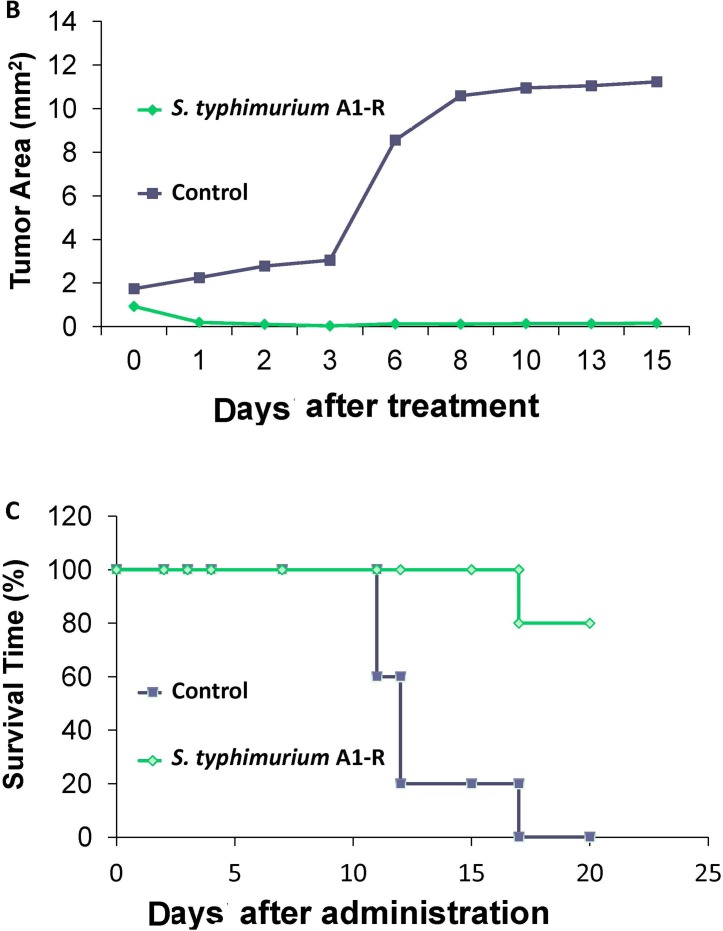
Efficacy of *S. typhimurium* A1-R on the growth of 4T1-RFP brain metastases and mouse survival (A) Nude mice with brain metastatic 4T1-RFP two weeks after surgical removal of orthotopic primary tumor. Mice were injected i.v. with *S. typhimurium* A1-R (5 × 10^7^ CFU in 100 μl PBS). Tumors were visualized with the Maestro fluorescence imaging system at indicated time points after infection. (Upper row): *S. typhimurium* A1-R treated mice. (Lower row): Control mice. (B) Growth curve of brain metastasis in control and *S. typhimurium* A1-R-treated mice (p<0.01). (C) Efficacy of *S. typhimurium* A1-R on survival of nude mice with 4T1-RFP breast cancer brain metastasis formed from orthotopic implantation and primary tumor resection. Survival of the *S. typhimurium* A1-R-treated animals was prolonged as shown by Kaplan Meier curves (p=0.005)

Nude mice with brain metastasis from 4T1-RFP high-metastic variants two weeks after surgical resection of the orthotopic primary tumors were injected directly into the tail vein with *S. typhimurium* A1-R (5 × 10^7^ in 100 μl PBS). Brain metastasis was visualized with the Maestro fluorescence imaging system at indicated time points after infection (Figure 4). *S. typhimurium* A1-R arrested brain metastasis growth (*S. typhimurium* A1-R-treated: 0.16 mm^2^ ± 0.15) compared to the untreated control mice in which the brain metastasis continued to grow (11.23 mm^2^ ± 3.5) at day 10 (Students *t-test*, p < 0.01) (Figure 4).

### Efficacy of *S. typhimurium* A1-R on survival of mice with 4T1-RFP breast cancer brain metastasis

Efficacy of *S. typhimurium* A1-R on survival of nude mice with 4T1-RFP brain metastases, formed after orthotopic transplantation of high-metastic variants and primary-tumor resection, was evaluated. The survival of treated animals was prolonged (*p*=0.005) (Figure 4C). All untreated mice with brain metastasis were dead by 17 days. In contrast, 80% of the mice were alive at day-21 post-initial treatment with *S. typhimurium* A1-R.

## DISCUSSION

Brain metastasis of 4T1 mouse mammary carcinoma was obtained after left cardiac ventricle injection or orthotopic injection in the mammary fat pad with high-brain-metastatic 4T1-RFP variants. Metastasis was extensive in the brain parenchyma. In the orthotopic model, surgical resection of the primary tumors increased the occurrence of brain metastasis. 4T1 metastasis in the brain of nude mice, was inhibited by *S. typhimurium* A1-R with subsequent prolongation of survival. This study shows the important potential of *S. typhimurium* A1-R therapy of breast cancer brain metastasis. The use of *S. typhimuirum* A1-R, a faculative anaerobe, has important advantages over obligate anaerobes such at *Clostridium novyi*, which does not appear to target metastasis since these bacteria seem to require i.t. administration [6, 24]. Breast cancer brain metastasis appears to be a promising target for clinical development of *S. typhimurium* A1-R.

Previously developed concepts and strategies of highly-selective tumor targeting [25-32] can take advantage of the *S. typhimuium* A1-R tumor targeting described in the present report.

## METHODS AND MATERIALS

### Mice

Athymic nude mice (*nu/nu*) mice (AntiCancer Inc., San Diego, CA), 6-8 weeks old, were used in this study. Nestin-driven green fluorescent protein (GFP) (ND-GFP) transgenic C57BL/6 nude mice (AntiCancer, Inc.) expressing GFP under control of the nestin promoter were also used [33-36]. All animal studies were conducted with the AntiCancer Institutional Animal Care and Use Committee (IACUC)-protocol specifically approved for this study and in accordance with the principals and procedures outlined in the National Institute of Health Guide for the Care and Use of Animals under Assurance Number A3873-1.

### Cell line

4T1 murine breast cancer cells expressing red fluorescent protein (RFP) were used as described previously [37]. The cells were cultured in DMEM medium.

### Selection of 4T1-RFP cells that preferentially metastasize to brain

4T1-RFP breast cancer cells (1.0 × 10^6^/100 μl) in serum-free solution (100 μl) were injected slowly into the right second mammary gland underneath the nipple (see below for details). When the average tumor volume reached approximately 500-600 mm^3^, the primary tumor was removed on day 14 after tumor implantation. Brain metastases were harvested when the mice became moribund. Cancer cells from the brain metastasis were cultured, amplified and then injected into the left cardiac ventricle of mice to generate brain metastases again. After 4 cycles of selection, 100% of the mice died of brain metastasis. There were no visible metastases to other organs (such as bones, lungs) within 1 month after injection.

### Mammary fat pad orthotopic injection of 4T1-RFP

Nude mice were anesthetized i.m. with a mixture of ketamine, acepromazine and xylazine (0.03 ml). 4T1-RFP breast cancer cells (1.0 × 10^6^/100 μl) in serum free solution (10 μl) were injected slowly into the right second mammary gland (underneath the nipple). The needle holes were pressed in order to prevent any cancer cells overflowing and seeding at the incision site.

### Surgical resection of the orthotopic primary tumor

The average tumor volume reached approximately 500-600 mm^3^ by day 14 after tumor implantation, at which time the primary tumor was resected. The animals were anesthetized with the mixture of ketamine, acepromazine and xylazine and the tumors were resected. Wounds were closed with 6-0 surgical sutures (silk). All procedures of the operation described above were performed with a 7× MZ6 magnification microscope (Leica Deerfield, IL) under HEPA-filtered laminar flow hoods.

### Preparation of *S. typhimurium* A1-R

GFP-expressing *S. typhimurium* A1-R was grown overnight in LB medium and then diluted 1:10 in LB medium. Bacteria were harvested at late-log phase, washed with PBS, and then diluted in PBS [10].

### Imaging

The OV100 Small Animal Imaging System (Olympus) containing an MT-20 light source (Olympus) and DP70 CCD camera (Olympus) [38], IV100 laser scanning microscope (Olympus) [39], FV1000 laser scanning confocal microscope (Olympus) [40] and the Maestro fluorescence imaging system (CRi, Caliper, Perkin-Elmer Inc., Hopkinton, MA) were used.

### Imaging of 4T1-RFP brain metastasis formation and *S. typhimurium* A1-R treatment

Nude mice and ND-GFP transgenic nude mice [33-36], which express GFP in nascent blood vessels, were used for analysis of brain metastasis of 4T1-RFP. All mice were injected either orthotopically or into the left cardiac ventricle with 4T1-RFP cells that were previously selected for 4 rounds *in vivo* for high brain metastasis. The tumors and vasculature in the brain were imaged by laser-scanning confocal and stereo fluorescence microscopy at various time points. The mice were injected i.v. with *S. typhimurium* A1-R (5 × 10^7^ CFU in 100 μl PBS).

### Efficacy of *S. typhimuirum* A1-R on survival of mice with brain cancer metastasis

Nude mice (10) with brain metastasis were identified 2 weeks after surgical removal of the orthotopic primary tumor. Nude mice (10) were randomized into treatment and control groups. Each group had 5 nude mice. The 5 mice in group 1 served as untreated controls. The 5 mice in group 2 were treated with *S. typhimurium* A1-R (5 × 10^7^ CFU in 100 μl PBS) via the tail vein once a week for 3 weeks. All mice were used for survival determination for 21 days post initial treatment.

## DEDICATION

This paper is dedicated to the memory of A. R. Moossa, M.D.
